# A deep learning model for predicting risks of crop pests and diseases from sequential environmental data

**DOI:** 10.1186/s13007-023-01122-x

**Published:** 2023-12-14

**Authors:** Sangyeon Lee, Choa Mun Yun

**Affiliations:** 1 Department of Bio and Brain Engineering, Korea Advanced Institute of Science and Technology (KAIST), 291 Daehak-ro, Yuseong-gu, Daejeon, 34141 Republic of Korea; 2 Sherpa Space Inc., Daejeon, 34028 Republic of Korea

**Keywords:** Crop disease, Pest, Deep learning, Environmental data, Prevention

## Abstract

Crop pests reduce productivity, so managing them through early detection and prevention is essential. Data from various modalities are being used to predict crop diseases by applying machine learning methodology. In particular, because growth environment data is relatively easy to obtain, many attempts are made to predict pests and diseases using it. In this paper, we propose a model that predicts diseases through previous growth environment information of crops, including air temperature, relative humidity, dew point, and CO_2_ concentration, using deep learning techniques. Using large-scale public data on crops of strawberry, pepper, grape, tomato, and paprika, we showed the model can predict the risk score of crop pests and diseases. It showed high predictive performance with an average AUROC of 0.917, and based on the predicted results, it can help prevent pests or post-processing. This environmental data-based crop disease prediction model and learning framework are expected to be universally applicable to various facilities and crops for disease/pest prevention.

**Supplementary Information:**

The online version contains supplementary material available at 10.1186/s13007-023-01122-x.

## Background

Crop diseases and pests increase the cost of production by reducing the yield and the quality of crops and increase the required labor, also cause environmental damage due to the overuse of chemicals to treat pests [1]. They are one of the major factors threatening food security, which causes about 15–40% of global grain yield loss [2–4]. In particular, powdery mildew, a significant pest and disease of strawberries covered in this paper, results in up to 70% yield loss [5, 6]. Also, gray mold, another major condition, generally damages 15–20% of strawberries and more than 50% in severe cases [7]. Pesticides used to prevent this are also used in trillions of US dollars, of which only 0.1% kills the target, and the rest is absorbed and distributed into the environment and causes pollution [8, 9]. In order to minimize the economic and environmental damages caused by these pests, several attempts have been made to predict pests [1, 10]. These disease modeling attempts create several mathematical models that explain the occurrence and development of pests by various factors and use it to predict the occurrence and severity of pests [11, 12], understand eases and pests reduce the yield and quality of crops, increase costs due to increased labor for pest treatment, the development of disease [13–15], It is used for assisting tactical decision making [16–18].

The major factors of pest modeling include the growth environment of crops, such as temperature, humidity, and precipitation. Among various factors that affect the occurrence of pests and diseases, the growing environment of crops is known to have a significant influence [1, 10, 19], and is often used for modeling because it is easier to collect and manage than other factors, such as host genomics [20], host nutrients [21], and microbial pathogen [22]. For example, A-scab [11], a model that predicts *Venturia inaequalis* infection in apples, calculates several intermediate variables using environmental variables of air temperature, rainfall, relative humidity, and leaf wetness and builds the criteria in the flow diagram. The presence or absence of pests is predicted corresponding to the flow diagram. INDIGOBLAST, a late blight prediction model of potatoes, also predicts pest occurrence by calculating the overall late blight severity index using thermal suitability and humidity suitability index calculated by temperature and humidity recordings [23].

These conceptual modeling methods have the advantage of being able to interpret predictions such as the underlying biological mechanism through [10] model. However, these models use many variables to predict pests and diseases, and a deep understanding of both target pests and crops is essential in integrating the variables. As a result, a new model must be built or adjust existing models heavily if the target crop or pest and disease for the model application are changed. However, creating models for an extensive range of crops and pests is impossible. The data-driven method has been actively studied because it can use large-scale and large-capacity complex data thanks to the recent development of big data and deep learning technology [24, 25]. Environment data [24, 26, 27] and image data [25, 28–30] are expanding to multi-modal [31]. Among various types of data, growing environment data is easy to collect on a large scale because the hardware for data collection is relatively easy to obtain and can be easily collected through devices installed in facility farms. To our best knowledge, there is still no model that predicts or forecasts the pest infestations of crops only with the growth environment information of crops in a universal situation by applying deep learning methodology.

In this paper, we propose a deep learning-based predictive modeling method applicable for preventing disease and pest infestation by predicting them through crop growth environmental data and calculating a linear risk score leading to a normal-infestation state. A prediction model was built with growth environment data collected from multiple facilities to show that the methodology is universally applicable regardless of the diversity of facility farm facilities, the diversity of crops, and target pests.

## Results

**Fig. 1:**
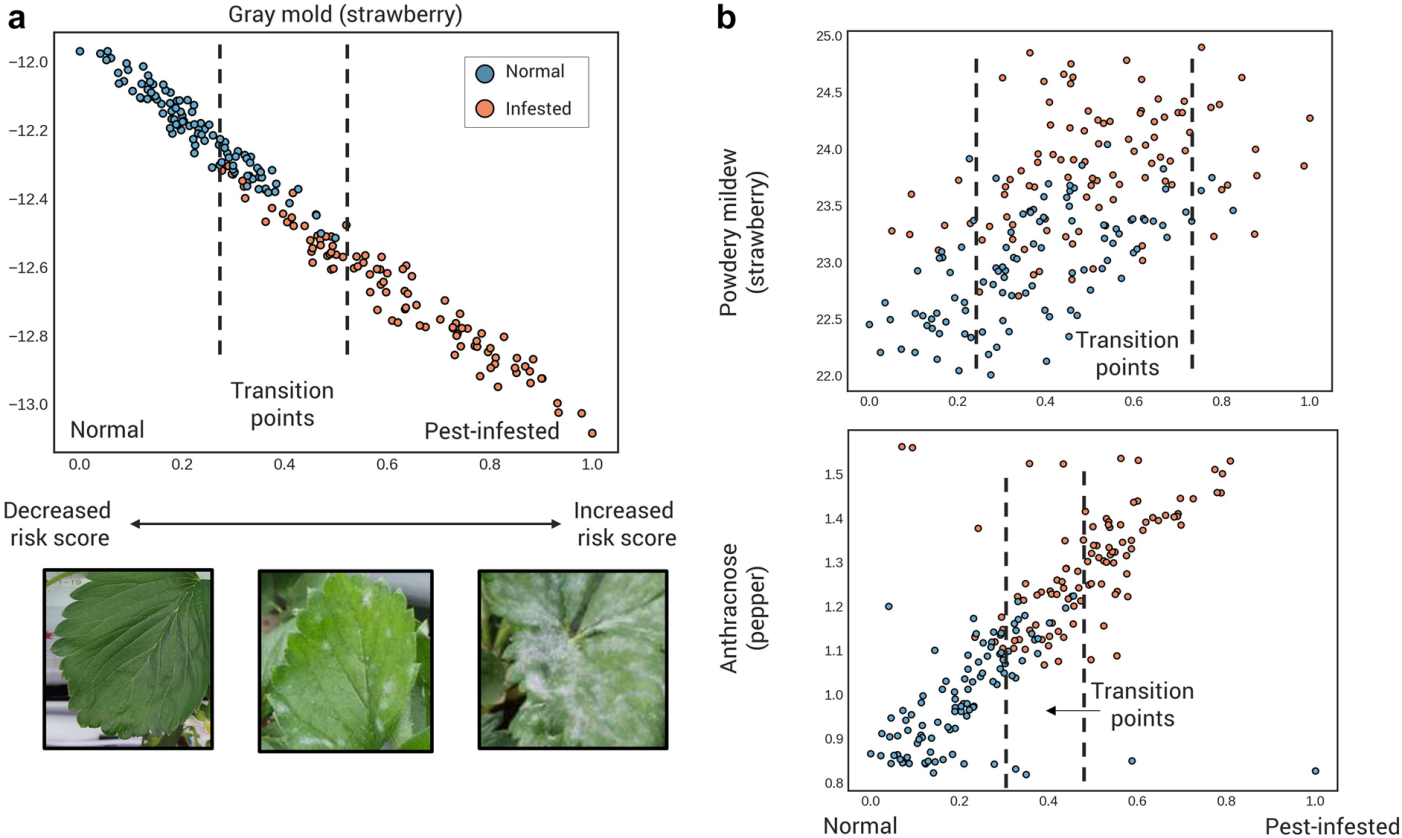
Two-dimensional embeddings of test data. Two-dimensional representations of test data and assumed transition stages of **a** strawberry gray mold data, **b** strawberry powdery mildew data, and **c** pepper anthracnose data. And **a** examples of strawberry leaf images of data located in normal, transition point, and pest-infested each

For most of the crops and pests used in this study, normal and infested crops were sorted with the status in the latent space using the test dataset (Fig. 1 and Additional file 1: Fig. S1). Data points tend to be classified and aligned from normal to infested clusters from coordinates α = 0 to α = 1. Due to the loss function used in the model, it was trained to gather normal points near α = 0 and the pest points near α = 1 on the latent space. The similarity of the cumulative environmental features is also represented by the distance between the points. In particular, there is a section in which normal and infested samples are mixed, representing the transition stage. It is the point that many samples are changing their status from normal to infested state. In the transition stage, the number of infested crops gradually increases with the change in the growth environment. It shows that it can be used as a model that predicts disease onset by increasing the risk score before the outbreak because it gives a continuous risk score rather than a binary classification result.

**Fig. 2:**
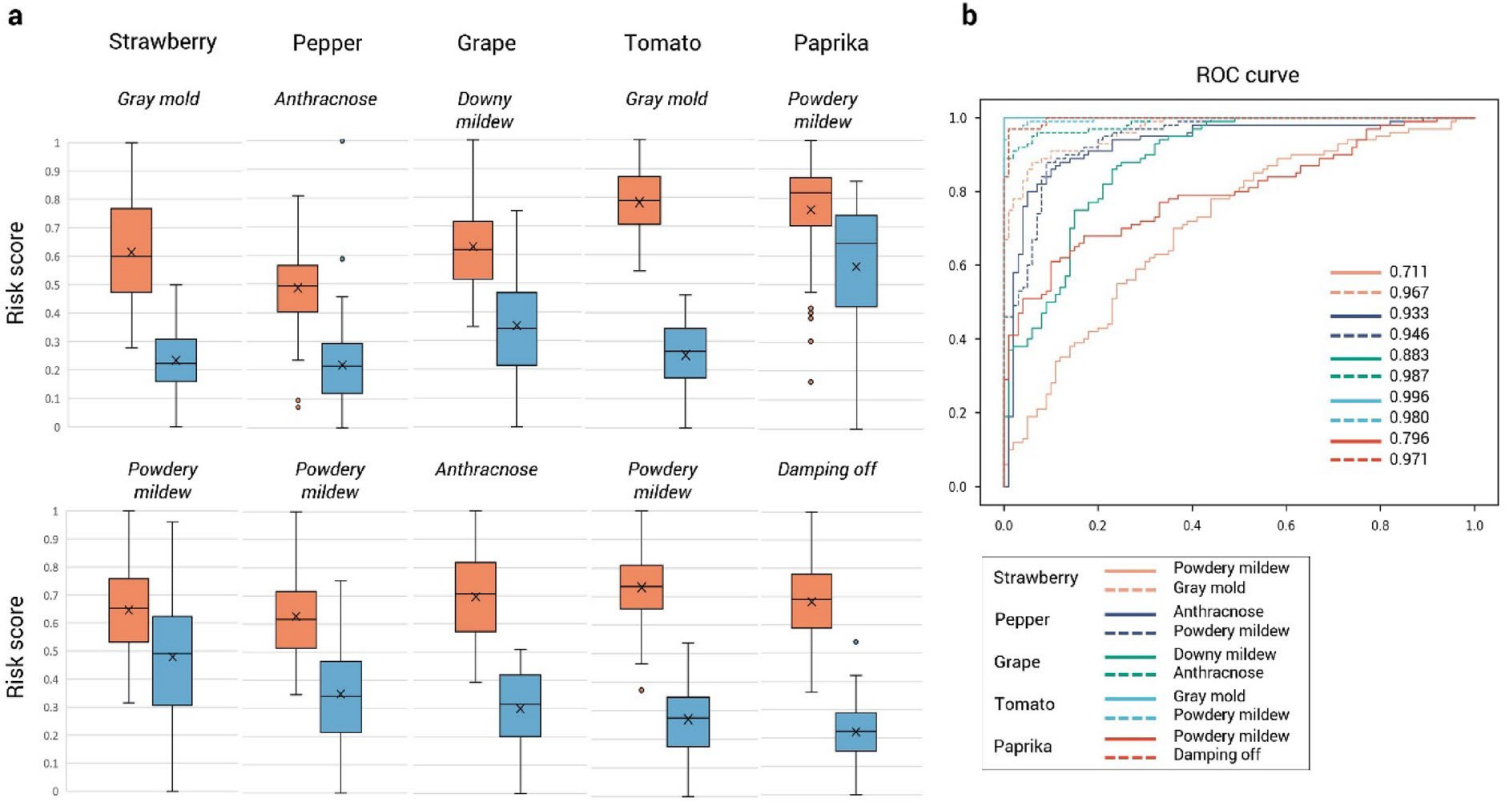
Risk score distributions and prediction result. **a** Risk score distributions of normal and infested samples per crop and pest. **b** ROC curve and AUROC of prediction result for every crops and pests. The color of the curve represents the type of crop and the shape of the line represents the type of pests for each crop

We tested how well the condition of the crop can be predicted from the accumulated environmental data using the risk score from the latent space. In all cases, the result shows that the risk score was significantly higher in the infested samples than in the normal cases (p < 0.05), with some overlaps in the transition stage (Fig. 2a). Normal and infected samples are very well distinguished from a gray mold of strawberry and tomato and from paprika, while widely overlapped in some cases, such as powdery mildew. We evaluated the crop disease prediction model using the ROC curve (Fig. 2b). The risk score was used as a rank to classify the crop's status and draw the ROC curve. The average of AUROC was 0.917, indicating that the model could classify the pests with high performance. In particular, the classification performance is consistent with the overlapping of risk scores. It is shown that the classification performance came out low in the case of powdery mildew because the risk scores of normal and infested samples overlap a lot. The low prediction performance and high overlap of risk scores can be interpreted that there are other influential factors in addition to the acquired environmental variables affecting the outbreak of the pest infestation. From the data science perspective, the model finds the relationships between the changes in growth environments and the outbreak of pests and diseases on the linear axis between 0 and 1. It learns how environmental differences lead to differences in risk scores. The overlaps occur if it is hard to explain the outbreak with only the environmental factors we used. Environmental variables such as solar radiation and relationships between host and fungi can be considered in further to improve the performance of the model [32, 33]. Experimental settings can be adequately adjusted for individual crops, pests, and diseases to alleviate the overlaps. Settings like two days of time window used in this study may be insufficient to train the onset of some rapid pests like powdery mildew [34, 35]. This problem can be alleviated by closely monitoring the growth environment or adjusting the number of sampling times while generating the training data.

We further investigated the relationship between disease severity and risk score. Using AI-hub's paprika damping off data, which includes the disease severity index, the predicted risk score is shown for each observation (Fig. 4a). The model is trained by a binary label, but it is shown that the risk score tends to increase as the severity of the disease increases. It shows that the prediction model can find a relationship between the exposed environment of crops and the occurrence and development of pests and diseases. However, since the difference in risk scores between infestation stages is small compared to the difference between normal and infested samples, it is crucial to maintain crops in a normal state by controlling the facility's environment before the transition point. Unlike binary classifiers, the prediction model uses risk scores as a surrogate measure for infestation prediction and can help tactical decision-making for pest prevention and management as a descriptive model. In order to test the possibility of a decision-making assistant for crop growth environment management, changes in the latent space were observed while adjusting the environmental data for one of the test data of strawberry gray mold and powdery mildew in the plant disease-causing dataset (Additional file 1: Fig. S3). The model can be used to prevent pests and diseases by managing the environment to the direction of maintaining a normal state by lowering the risk score caused by the movement of data point in the latent space led by the change in the growth environment.

**Fig. 3:**
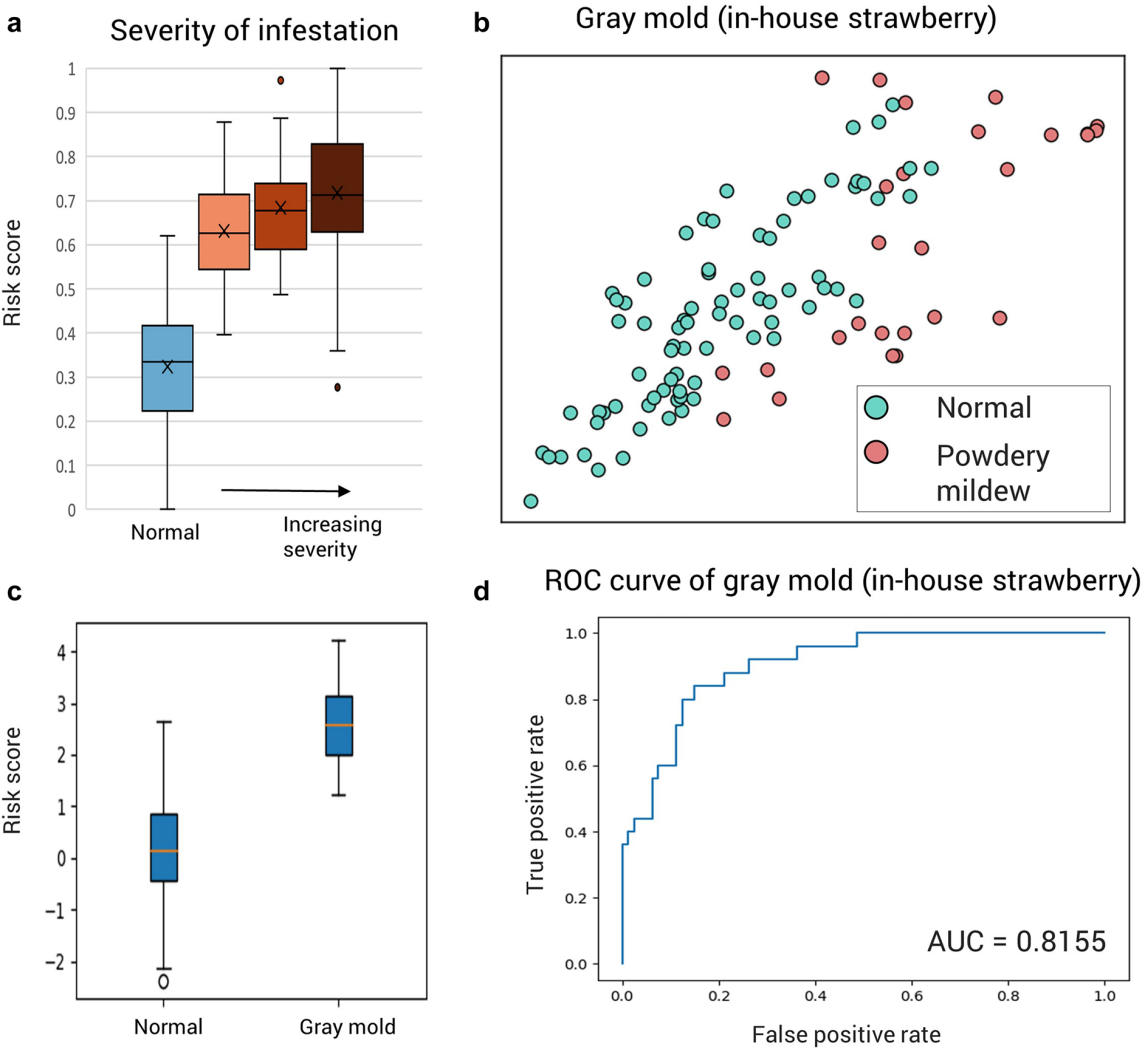
Validation of the trained model. **a** Risk score distributions corresponding to the severity of observed infestation. Severity of paprika damping off increases from orange plot to brown plot. **b** Validation of gray mold prediction model using independent in-house dataset (latent space). **c** Risk score distributions of in-house samples; gray mold and normal. **d** Model evaluation of in-house gray mold prediction

Also, we tested our model with the in-house dataset, which contains strawberry gray mold data, to show the model trained with the plant disease-causing dataset can be applied to other datasets. The test data was embedded in the latent space and showed that the normal and infested samples were well aligned (Fig. 3b). The risk score distributions of the normal and infested samples were also consistent with the test results of the plant disease-causing dataset. Also, the prediction performance was evaluated with AUROC, and shows decent performance with AUROC of 0.8755 (Fig. 3c, d). However, the range of predicted risk scores was different. It may be caused by the difference in species of strawberry or fungi and environmental variety due to the facility, such as soil quality. This means some degree of overfitting occurs because input data cannot include every influential variable for the pest. Alignment corresponding to the risk of pest infestation is possible even with the pre-trained model, but the scale of the risk score is hardly matched. It shows that the fine-tuning stage is essential before applying the model to the individual facility.

## Discussion

In this paper, we created a deep learning model to predict the presence or absence of pests using crop growth environmental data. It was shown that it is possible to control the environment for early diagnosis and prevention of pests and diseases through continuous environmental data monitoring. To this end, public and in-house data regarding crops' state and growth environment were collected. Moreover, to deal with large-scale heterogeneous data, we built an independent model for variables such as types of environmental variables, measurement intervals, and types of crops. Through this, it is possible to predict the status of a crop from its environmental data of the past two days. At the same time, the risk score can be extracted through the latent space. Unlike the binary classification, the risk score revealed a transition stage connecting the normal and disease groups. Early diagnosis and prediction before the outbreak will be possible during the transition stage. If a crop is located in the transition stage before the occurrence of pests and diseases, it is possible to prevent them by controlling the environment to reduce the risk score. This paper showed that environmental control is possible by reducing the risk score and moving the data close to the normal state by observing the pattern in the latent space that appears while changing the environmental variables from the point of interest through simulation.

Still, some challenges must be overcome to apply this model to existing facilities or farms. Many factors affect crop pests and diseases compared to the environmental variables measured in this study. There are plenty of factors that are not included in this study but are known to influence the growth of crops, such as soil components, soil pH, microbial properties, precipitation, and air current. In addition, in open-farm conditions, some variables are hard to quantify for data collection such as weather events, pollinators, and weeds [36, 37]. For example, compared to indoor farms that can artificially control many environmental variables, open-field farms have many variables, so it is difficult to train a model considering all of them. It is highly required to develop a multimodal monitoring system that collects high-resolution environmental data by quantifying individual features, as various environmental factors listed above are known to affect crop growth, diseases, and pests. In addition, considering relationships between different diseases or pests and optimal crop growth conditions, the model needs to be more complex. Resilience and resistance against a harsh environment and optimal growth environment are different, corresponding to the growth stage of crops [38]. Considering this, we can expect an increase in performance and practicality by adding temporal factors or the acquired growth stage data of target crops in the aspect of the life cycle to the training phase. The construction of a database suitable for these tasks must be preceded.

However, this model is highly versatile as it can be optimized and applied in various situations by adjusting parameters such as the type of environmental variable or the period of environmental recording. The resolution, performance, and usability of the model can be increased with additional sensors on various environmental variables. In addition to environmental data, various time-series information that can be acquired by sensors can be utilized in this method. In an experimental environment where environmental variables can be finely controlled, this model can be used for physiological studies such as crop-pest interactions and crop reactions to environmental changes. In addition, in well-established facility farms, early management or prevention of pests and diseases can help improve crop quality, increase yield, and reduce required labor.

## Methods

A large-scale dataset containing data on whether crops were infested with pests and diseases while growing and environmental information recorded during that period is needed to create a model that predicts the condition of crops from their growing environments. We used large-scale public database, ‘plant disease-causing dataset’ for the data, reduced the dimensionality of the data, and built a model to predict the condition of crops using deep-learning methodology.

### Data acquisition

Large-scale data were collected to build a model for predicting the occurrence and risk of pests and diseases with crop growth environment data. A public database named 'plant disease-causing dataset' provided by AI-hub is used. The dataset is data on disease and disease according to the environment of crops grown in several facilities. It provides disease outbreak data and cumulative environment data for at least 48 h before the observation time (Fig. 1a). Environment data includes at least temperature and humidity data and additionally collects CO_2_, solar radiation, dewpoint, etc., depending on the facility equipment. We acquired environment and pest infestation data from the database for five crops and two pest infestations per crop. The number of data used in this study is shown in Table 1. The collected data includes various environmental data, temperature and humidity for all data, and other environmental data depending on the facility. With temperature and humidity recordings, grape data contains dewpoint data, and strawberry, pepper, and tomato data contains dewpoint and CO_2_ concentration, while paprika data contains solar radiation measures. The diversity of data is a problem that inevitably arises due to the environmental information collection facilities of actual farms or the diversity of crop types.

**Table 1:** Data statistics acquired from the plant disease-causing dataset

Crop	Disease	# of data
Strawberry	Gray mold (*Botrytis cinerea*)	16,000
	Powdery mildew (*Spharotheca humuli*)	10,000
Pepper	Anthracnose (*Colletotrichum acutatum*)	13,000
	Powdery mildew (*Leveillula taurica*)	12,000
Grape	Downy mildew (*Plasmopara viticola*)	8500
	Anthracnose (*Colletorichum fructicola*)	2500
Tomato	Gray mold (*Botrytis solan*)	12,000
	Powdery mildew	10,000
Paprika	Powdery mildew (*Leveillula taurica*)	26,000
	Damping off (*Rhizoctonia solini*)	1000

In addition, as in-house data for validating the model trained using AI-hub, data on strawberry gray mold measured from November 2020 to May 2021 were collected. During the period, air temperature, solar radiation, relative humidity, and dewpoint of the strawberry facility were collected as environmental information every 10 min, and the percentage of infested strawberries in the bed was measured. Unlike the plant disease-causing dataset that contains binary crop condition information, the in-house data is obtained as a continuous value because the percentage of diseased crops out of the total crops is measured.

### Prediction model

**Fig. 4:**
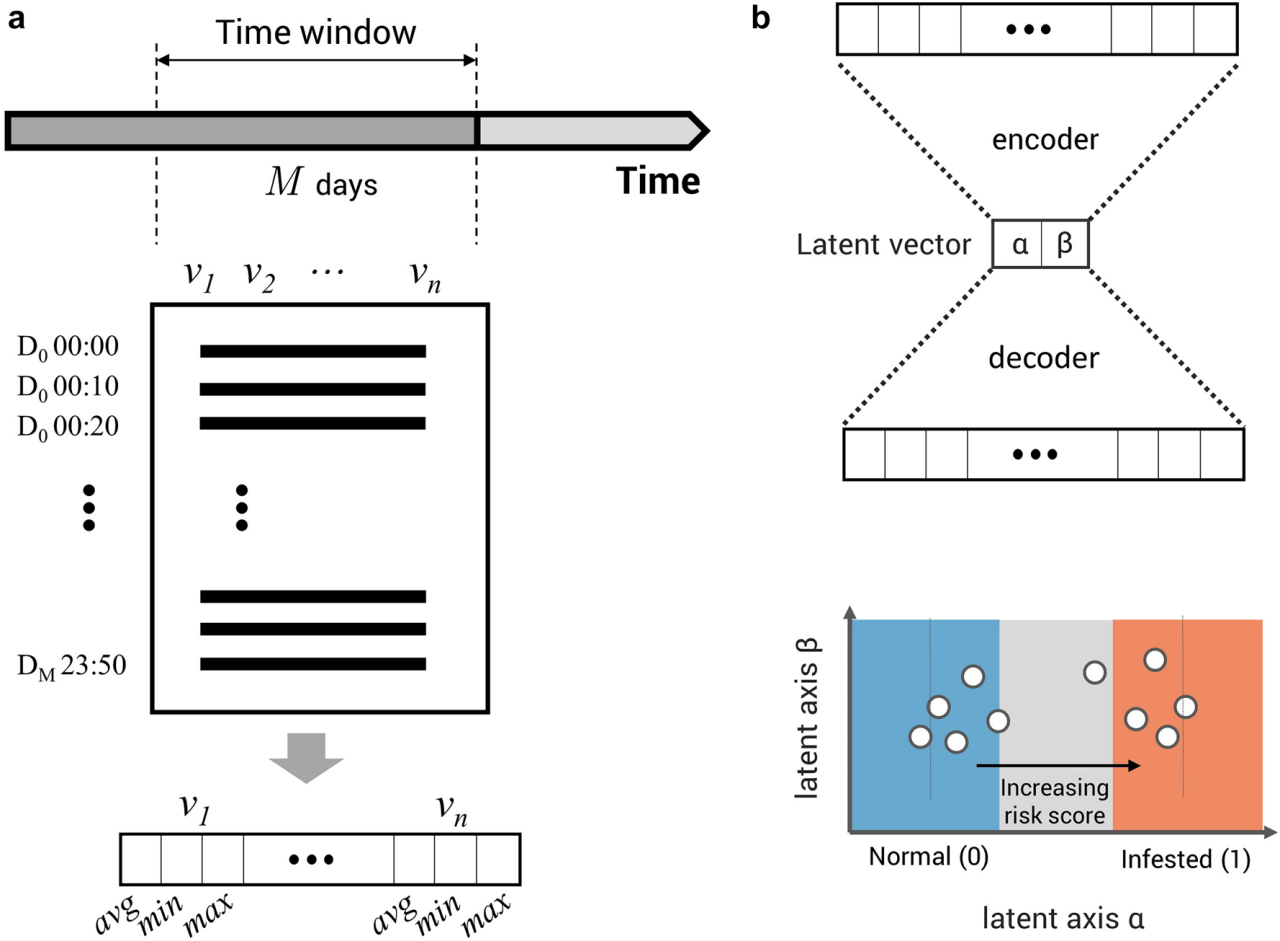
A schematic of data acquirement and the prediction model. **a** A simplified representation of the format of data and transformation to one-dimensional vector obtained during the data measurement period. *M* is set to 2 as we acquired recordings of 48 h. **b** schematic of autoencoder structure and optimal embeddings to the latent space

A deep learning-based methodology was applied to build a model that predicts crop conditions at a specific point in time through past growing environments [39]. The autoencoder-based model was developed to predict the condition of crops with environmental data measured for the past two days from the forecasting point (Fig. 4b). The model used in this study takes an input vector representing two days of observations. Two days of cumulative environmental data were converted into a one-dimensional vector to make it an input for the model. For all environmental variables included in the data, the average, minimum, and maximum values of the measured values during the period are calculated, and all of these are concatenated to form a one-dimensional vector representing that period of observation. As a result, for a facility measuring n environmental variables, an input vector of length 3*n* is created (Fig. 4a).

The prediction model consists of an encoder composed of two layers with the same length as the input vector, a decoder having the same structure as the encoder, and a two-dimensional latent vector. The model was developed in a PyTorch environment based on modified loss functions suggested by the original paper [39]. Ordinary autoencoders are trained using the error between the input vector and the reconstructed vector as loss, but in this study, how well it predicts the label representing the state of the crop is added to the loss function (Additional file 1: Fig. S2). In the training phase, the mean squared error between the input and reconstructed vectors and the distance between the reference point on the alpha axis corresponding to the label was added and used as a loss function to make the normal and pest data points agglomerate respectfully. We set the reference point alpha = 0 for normal data and alpha = 1 for pest data. Since the data points are aligned along the alpha axis, we get the continuous value of the alpha coordinate of the data point, which can be considered as a risk score. So the prediction model can be used as an autoencoder-based regression model, although we trained the model using binary-labeled data. We trained a model in AI hub data to classify environmental vectors based on labels such as the presence or absence of crop diseases or pests. In all cases, we used 80% of the data as training data and the rest as test data. In the learning process, the learning rate, weight decay, and epoch were optimized for crop and pest respectively, while batch size is fixed to 64. After the hyperparameter optimization, five-fold cross validation was performed. The risk scores from each trial was used for further analysis.

### Supplementary Information

**Additional file 1: Figure S1.** Embedded points of test data on two-dimensional latent space. **Figure S2.** Change of loss during the training. **Figure S3.** Movement of data points with simulation.

## Data Availability

The dataset used in this study is a public dataset with limited access that can be used after approval by the National Information Society Agency (NIA). Details of the dataset can be found on the AI-Hub website. (https://aihub.or.kr/aihubdata/data/view.do?currMenu=115&topMenu=100&aihubDataSe=realm&dataSetSn=525) The in-house data of this study are available from the corresponding author, C. M. Yun, upon reasonable request.
