# Correction: A deep learning model for predicting risks of crop pests and diseases from sequential environmental data

**DOI:** 10.1186/s13007-024-01140-3

**Published:** 2024-02-04

**Authors:** Sangyeon Lee, Choa Mun Yun

**Affiliations:** 1 Department of Bio and Brain Engineering, Korea Advanced Institute of Science and Technology (KAIST), 291 Daehak-ro, Yuseong-gu, Daejeon, 34141 Republic of Korea; 2 Sherpa Space Inc, Daejeon, 34028 Republic of Korea

**Correction to: Plant Methods (2023) 19:145**

10.1186/s13007-023-01122-x

In this article [1], the e-mail address of the corresponding author, Choa Mun Yun was incorrectly given as sangyeonlee230@gmail.com but should have been cmunyun@sherpaspace.co.kr.
