# Supplementary material for: A deep learning model for predicting risks of crop pests and diseases from sequential environmental data
Source: Plant Methods. 2023 Dec 14;19:145. doi: 10.1186/s13007-023-01122-x (PMC10720067; doi:10.1186/s13007-023-01122-x)
Supplement: Supplementary file 1 — Additional file 1: Figure S1. Embedded points of test data on two-dimensional latent space. Figure S2. Change of loss during the training. Figure S3. Movement of data points with simulation. [file 13007_2023_1122_MOESM1_ESM.docx]

Additional file 1: A deep learning model for predicting risks of crop pests and diseases from sequential environmental data

*Sangyeon Lee^1^, and Choa Mun Yun^2*^*

1) Department of Bio and Brain Engineering, Korea Advanced Institute of Science and Technology (KAIST), 291 Daehak-ro, Yuseong-gu, Daejeon, 34141, Republic of Korea

2) Sherpa Space Inc., Daejeon, 34028, Republic of Korea

* Corresponding Author

E-mail: [sangyeonlee230@gmail.com](mailto:sangyeonlee230@gmail.com), [cmunyun@sherpaspace.co.kr](mailto:cmunyun@sherpaspace.co.kr)

Keywords: Strawberry Disease, Pest, Deep Learning, Environmental data, Prevention


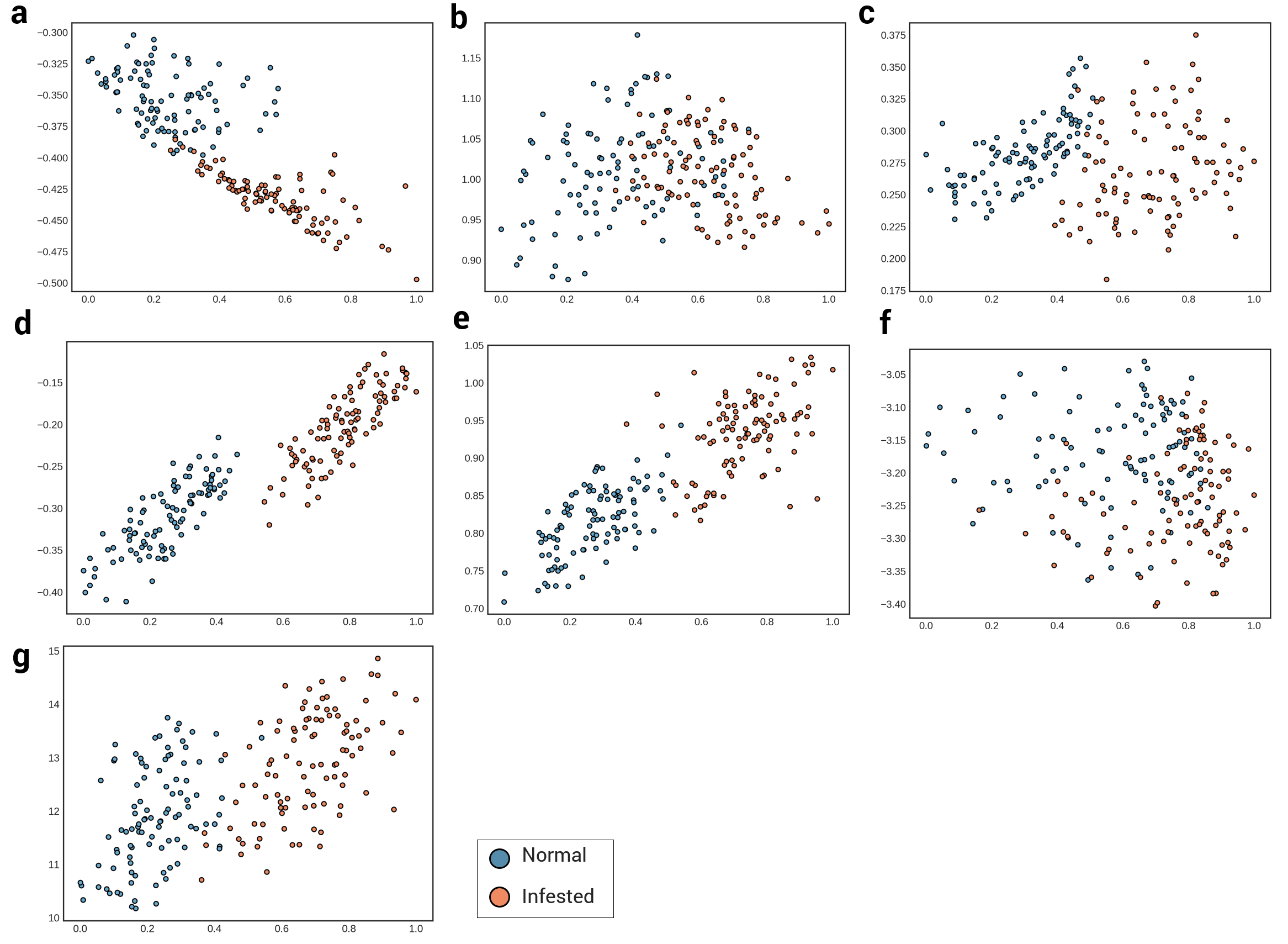


**Figure S1. Embedded points of test data on two-dimensional latent space.**

**a) Powdery mildew (pepper), b) downy mildew (grape), c) anthracnose (grape), d) gray mold (tomato), e) powdery mildew (tomato), f) powdery mildew (paprika), and g) damping off (paprika).**


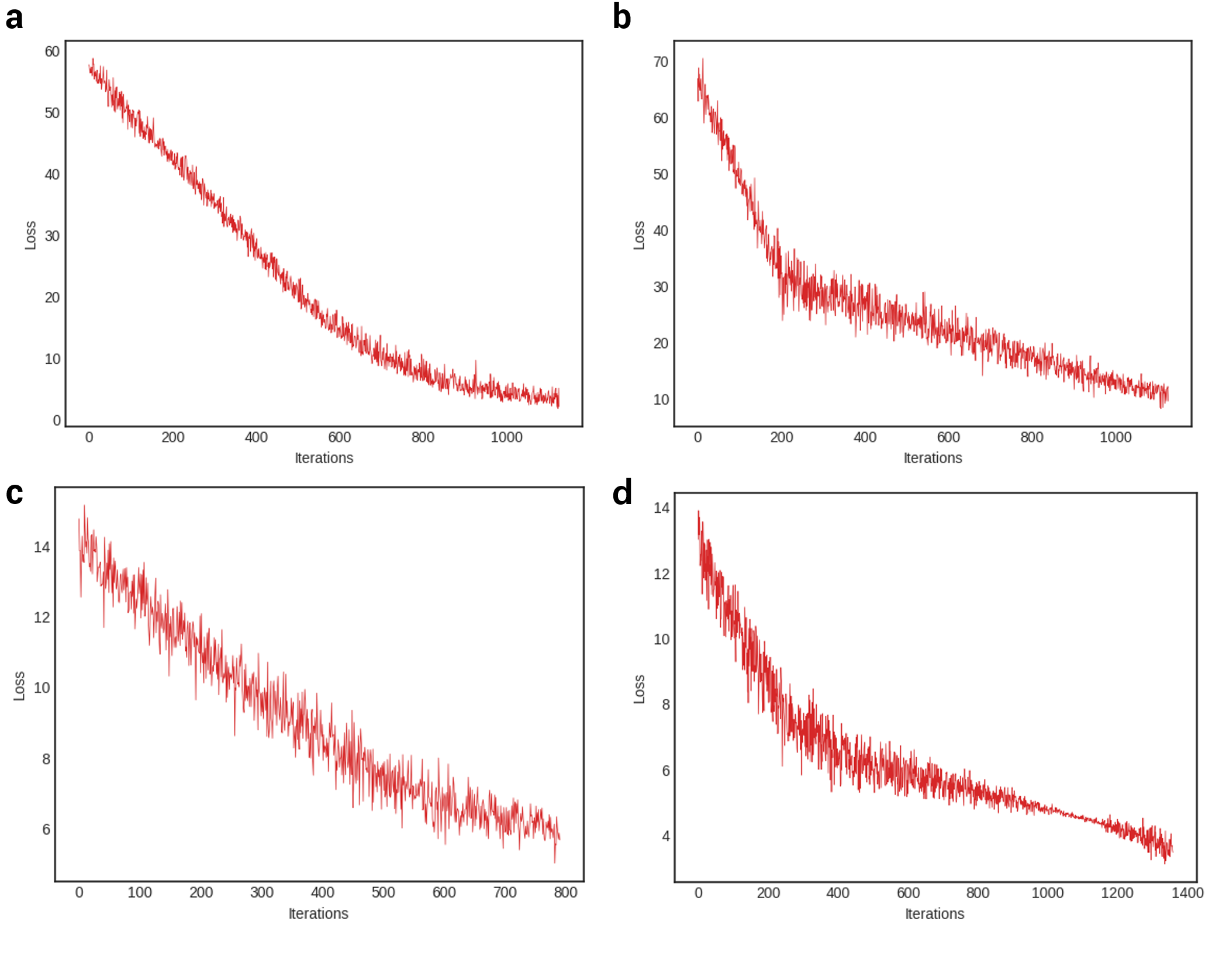


**Figure S2. Change of loss during the training.**

**Plots shows the examples decrease of global losses in training phase. a) strawberry gray mold, b) strawberry powdery mildew, c) pepper anthracnose, d) tomato gray mold. As powdery mildew shows lower prediction performance than others, the absolute value of loss is higher than in other cases.**


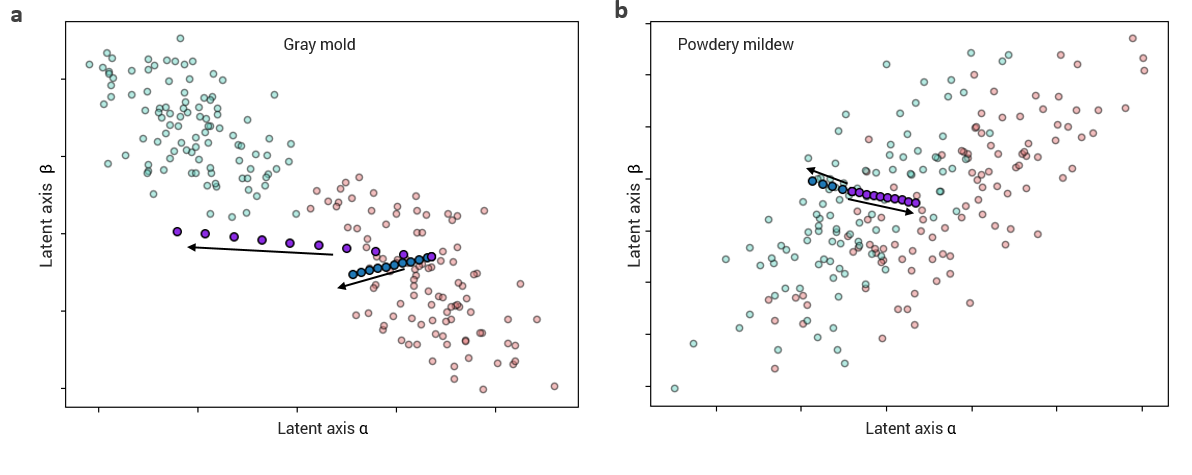


**Figure S3. Movement of data points with simulation**

**Changes of data points in the latent space followed by an environment change in a) gray mold data and b) powdery mildew data. Transparent points represent normal points (green) and disease points (red). Purple and blue points represent the results of generated data from the simulation. 20 virtual data samples were created from the original data point. Ten of them were generated by decreasing the relative humidity by 1%p to 10%p (purple points). The other then points are generated by decreasing the soil temperature by 0.1°C from the original (blue points). Arrow indicates that the direction of movement from the original data.**
